# Alterations of the alpha rhythm in visual snow syndrome: a case-control study

**DOI:** 10.1186/s10194-024-01754-x

**Published:** 2024-04-08

**Authors:** Antonia Klein, Sarah A. Aeschlimann, Frederic Zubler, Adrian Scutelnic, Franz Riederer, Matthias Ertl, Christoph J. Schankin

**Affiliations:** 1 Department of Neurology Inselspital, Bern University Hospital, University of Bern, Rosenbühlgasse 25, Bern, CH-3010 Switzerland; 2 Department of Psychology, University of Bern, Bern, CH 3010 Switzerland; 3 Neurocenter, Luzerner Kantonsspital, Lucerne, 6000 Switzerland

**Keywords:** Visual snow syndrome, Alpha rhythm, EEG, Thalamocortical dysrhythmia, Network disorder

## Abstract

**Background:**

Visual snow syndrome is a disorder characterized by the combination of typical perceptual disturbances. The clinical picture suggests an impairment of visual filtering mechanisms and might involve primary and secondary visual brain areas, as well as higher-order attentional networks. On the level of cortical oscillations, the alpha rhythm is a prominent EEG pattern that is involved in the prioritisation of visual information. It can be regarded as a correlate of inhibitory modulation within the visual network.

**Methods:**

Twenty-one patients with visual snow syndrome were compared to 21 controls matched for age, sex, and migraine. We analysed the resting-state alpha rhythm by identifying the individual alpha peak frequency using a Fast Fourier Transform and then calculating the power spectral density around the individual alpha peak (+/- 1 Hz). We anticipated a reduced power spectral density in the alpha band over the primary visual cortex in participants with visual snow syndrome.

**Results:**

There were no significant differences in the power spectral density in the alpha band over the occipital electrodes (O1 and O2), leading to the rejection of our primary hypothesis. However, the power spectral density in the alpha band was significantly reduced over temporal and parietal electrodes. There was also a trend towards increased individual alpha peak frequency in the subgroup of participants without comorbid migraine.

**Conclusions:**

Our main finding was a decreased power spectral density in the alpha band over parietal and temporal brain regions corresponding to areas of the secondary visual cortex. These findings complement previous functional and structural imaging data at a electrophysiological level. They underscore the involvement of higher-order visual brain areas, and potentially reflect a disturbance in inhibitory top-down modulation. The alpha rhythm alterations might represent a novel target for specific neuromodulation.

**Trial registration:**

we preregistered the study before preprocessing and data analysis on the platform osf.org (DOI: 10.17605/OSF.IO/XPQHF, date of registration: November 19th 2022).

**Graphical Abstract:**

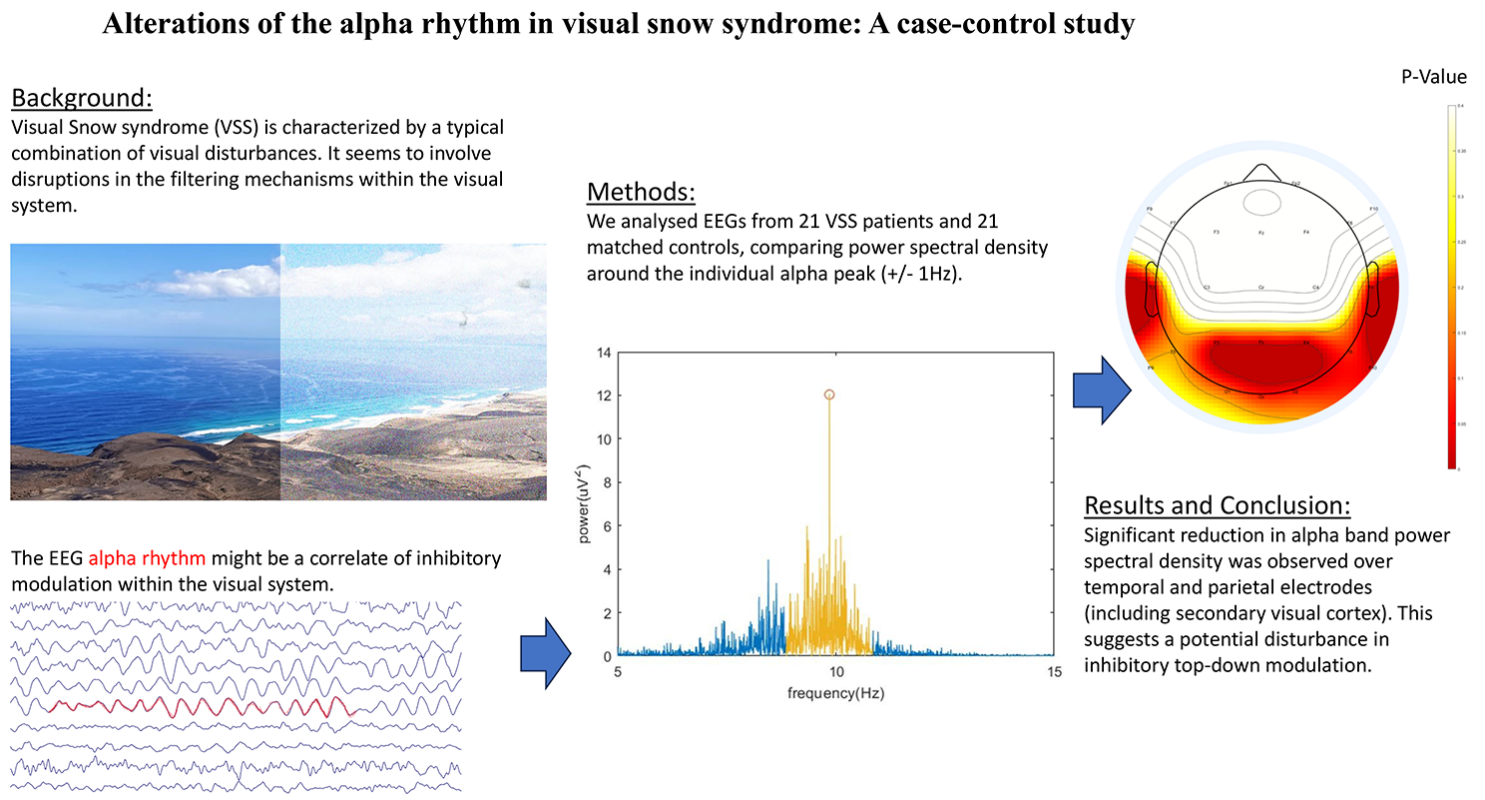

## Background

Visual snow syndrome (VSS) is associated with migraine and is characterised by the combination of persistent typical visual phenomena [1]. The name giving main symptom, “visual snow” is often described as a flickering across the entire visual field [2]. Case studies with patients with occipital, temporal or parietal brain lesions and acute development of VS suggest involvement of primary and secondary visual brain areas in the generation of this phenomenon [3–5].

The additional symptoms associated with VSS indicate hypersensitivity to both internal and external visual stimuli. These symptoms include photophobia (increased sensitivity to light) and enhanced perception of entoptic phenomena (physiological visual artefacts) [2]. Patients also have difficulties seeing under conditions that require increased contrast differentiation, such as at night (nyctalopia) [2]. They also describe perceiving objects repetitevly i.e. palinopsia [2], which can also occur in lesions of the parieto-occipital or posterior temporal lobe cortex [6].

In neuroimaging, individuals affected by VSS show functional and structural differences in various areas of the visual system, higher-order attentional networks and in the limbic system [7–11]. Electrophysiological findings suggest a disturbance of inhibitory processes over the visual cortex [12, 13], as well as disturbances in upstream processing [14].

Regarding regional oscillatory patterns, one group observed enhanced gamma frequency power over the primary visual cortex of a single patient, relative to healthy controls during visual stimulation [12].

Gamma oscillations might represent local encoding mechanisms reflecting the interplay of inhibitory and excitatory interactions at a cellular level [15–17]. In a magnetoencephalography (MEG) study, significantly increased gamma but no significant changes in alpha power over the primary visual cortex (V1) were detected a group of 18 participants with VSS. Moreover, there was a decreased alpha-gamma coupling (synchronisation), suggesting disturbances in the interactions between these frequency bands [18]. While gamma frequencies have been shown to correlate with regional activity patterns, alpha waves are thought to modulate local cortical activity on a larger scale, by orchestrating local gamma activity periodically [19, 20].

The alpha frequency is easily detectable in surface EEG due to its typical topographic distribution and slower oscillation range (8-13 Hz). It is typically recorded over occipital brain areas during resting wakefulness (eyes closed) [21].

The alpha rhythm appears to be more than just a correlate of “visual inactivity” during the resting state. Multiple studies have demonstrated an inverse correlation between visual detection performance and alpha amplitude at stimulus onset, indicating the involvement of alpha oscillations in visual filtering processes and possibly in the determination of perceptual thresholds [22, 23]. Furthermore, alpha power increases on the ipsilateral side of a target stimulus during a visual task, suggesting the suppression of task-irrelevant visual input as a form of guiding spatial attention [24, 25].

Since patients with VSS often report pronounced symptoms during resting wakefulness, we aimed at exploring the alpha rhythm at rest in patients with VSS. We hypothesised that patients with VSS would have, compared to control subjects matched for age, sex, and comorbid migraine, decreased power spectral density in the alpha band (alpha PSD) over the primary (primary hypothesis) or supplementary visual cortex (secondary hypothesis). Based on studies about migraine [26] and chronic pain [27], we also hypothesized a reduction of individual alpha peak frequency (IAF), i.e. the frequency corresponding to the individual peak in the alpha frequency range.

## Methods

The experimental procedures complied with the Declaration of Helsinki and were approved by the cantonal ethics committee. Patients with VSS were included based on the general consent procedure at our institution when they had received an EEG as part of their clinical routine. Clinical information was extracted from the patient’s files. All VSS patients had had at least one outpatient consultation at our headache clinic. We have preregistered the study on osf.org (osf.io/xpqhf). One patient who had given consent orally did not finally sign the general consent form and had to be excluded after the preregistration. So, we ended up including 21 participants per group and not 22 as preregistered. The characteristics of the patient group are shown in Table 1.

**Table 1:** Visual symptoms of patients with VSS

Symptoms	VSS (*n* = 21)	
Visual snow (%)	21	(100)
Palinopsia (%)	14	(66.6)
Enhanced entoptic phenomena (%)	20	(95.2)
Photophobia (%)	21	(100)
Impaired night vision (nyctalopia) (%)	13	(61.9)

The control group consisted of 21 individuals from our EEG database matched for age, sex, and migraine. We matched for these factors since migraine can influence the alpha rhythm [26, 28] and approximately two-thirds of individuals with visual snow syndrome also experience migraines [29]. Ageing is known to affect the alpha rhythm [30], and sex might also have effects on the EEG [31, 32].

The selection of controls was based on a thorough review of patient files, also considering comorbidities (listed in Table 2) and the reasons for receiving EEGs, which were: episodes of loss of consciousness retrospectively diagnosed as probable syncope (6/21), concentration problems (2/21), migraine (4/21), tiredness/insomnia (2/21), psychiatric symptoms (6/21), and vegetative symptoms (1/21). For controls, we excluded individuals with pathological EEGs (general slowing, moderate or severe focal slowing or epileptiform discharges), specific psychiatric disorders (psychosis, schizophrenia, bipolar disorder, drug intoxication), and brain lesions, with the exception of unspecific white matter lesions commonly observed in individuals with migraine [33]. Given the high prevalence of depression and anxiety among our patients with VSS, we included controls with a similar fraction of these disorders. However, exact matching was not feasible.

**Table 2:** Demographic and clinical characteristics of the patients

Characteristic	VSS (*n* = 21)		Controls (*n* = 21)	
Male sex – no. (%)	13	(61.5)	13	(61.5)
Migraine – no. (%)	14	(66.6)	14	(66.6)
Age – mean ± SD [yr]	33	± 9.6	33	± 11.1
Psychiatric comorbidities – no. (%)				
Depression/Anxiety	8	(38.1)	5	(23.8)
ADHD	1	(4.7)	1	(4.7)
Functional neurological disorders	2	(9.5)	3	(14.3)
Eating disorder	1	(4.7)	0	(0)
Obsessive-compulsive disorder	0	(0)	1	(4.7)
Previous recreational drug use	0	(0)	1	(4.7)
Medication – no. (%)				
None	12	(57.1)	13	(61.9)
SSRI/ SNRI/Tricyclic antidepressant	4	[19]	5	(23.8)
Anticoagulation	0	(0)	2	(9.5)
Antihypertensiva/Antidiabetics	1	(4.7)	2	(9.5)
Anti-Seizure-medication (Lamotrigine)	2	(9.5)	1	(4.7)
Calcium Channel blocker (Cinnarizine)	0	(0)	1	(4.7)
Lisdexamfetamindimesilat	1	(4.7)	0	(0)
Benzodiazepines (intermittent)	1	(4.7)	0	(0)
Intermittent THC (intermittent)	1	(4.7)	1	(4.7)
Other comorbidities – no. (%)				
Chronic pain syndrome	1	(4.7)	0	(0)
Chronic Leukaemia	1	(4.7)	0	(0)
OSAS	0	(0)	1	(4.7)
Polycythämia vera	0	(0)	1	(4.7)

### EEG acquisition

The EEGs were recorded on a NicoletOne-EEG-System in the EEG laboratory of the Sleep-Wake-Epilepsy-Centre using the 25 electrodes system recommended by the international neurophysiology society [34], with reference near Cz. The sampling rate was 250/s with each recording lasting 20 min. In the beginning, the patients were asked to close their eyes; it was regularly checked if they were awake.

The EEG files were visually inspected, and 8 epochs with 20s each with as few artefacts as possible (eyes closed/awake) were selected per patient. No patient recordings were excluded due to artefacts.

### Preprocessing

Preprocessing was done in EEGlab (Matlab version 2022b, EEGlab 2022.1 and 2023.0). In the first step, we filtered the data (Highpass 1 Hz, Lowpass 45 Hz), then we performed independent component analysis (Algorithm: “jader”) to remove blink, movement, and muscle artefacts. We referenced the EEGs to the retroarticular electrode (T9/T10).

### Data analysis

**Fig. 1:**
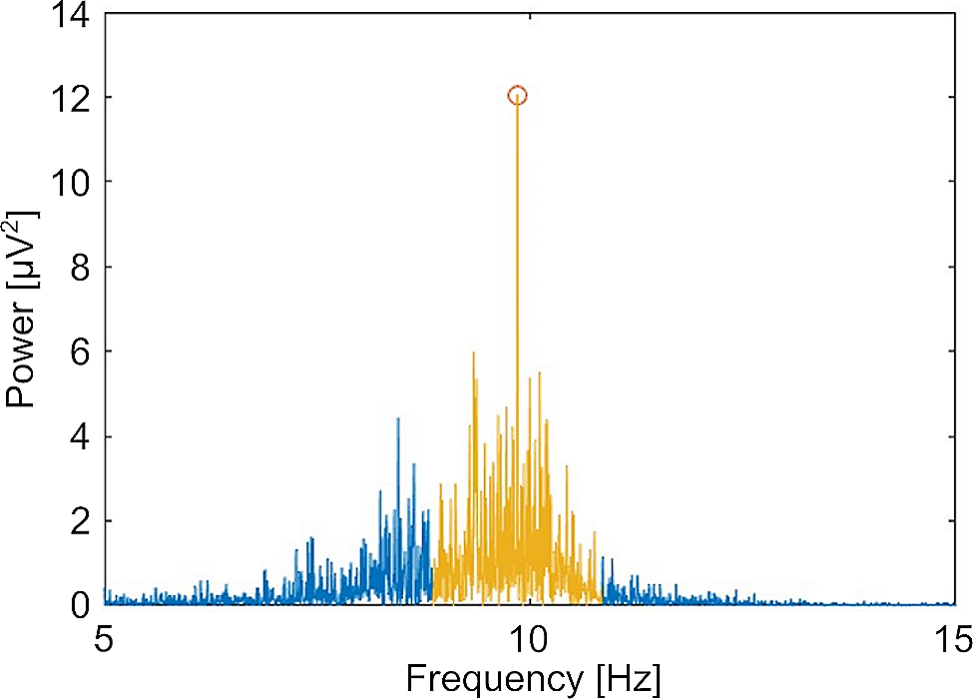
Visualization of the alpha PSD over the frequency spectrum (exemplarily shown for subject V1, electrode O1)

A Fast Fourier Transform (FFT) was used to compute the power spectrum of EEG signals. We then considered the individual main power peak within the alpha frequency range (8-13 Hz). We further computed the mean power spectral density (Fig. 1) within a range of ± 1 Hz around the IAF [31].

For visualisation of the spatial representation of the power spectral density in the alpha band (alpha PSD) across different electrodes we utilized the ‘topoplot’-function. Since the alpha PSD results were not normally distributed, as assessed with the Kolmogorov-Smirnov-test, we plotted the group medians for each electrode.

The distribution of the alpha PSD showed higher values in the control group compared to the VSS group extending beyond the occipital cortex into temporal and parietal areas. Therefore, we opted to conduct an additional exploratory analysis comparing the alpha PSD over these brain areas.

### Statistics

We performed a series of non-parametric statistics to compare patients with VSS and controls to detect between-group differences in alpha PSD and IAF. First, the omnibus Kruskal-Wallis-test was used. When significant, defined by *p* < 0.05, we used the Wilcoxon-Mann-Whitney-test as post-hoc test, corrected for multiple comparisons using the false discovery rate (FDR) approach.

We also compared the IAF results over the primary visual cortex (O1 and O2). Since the IAF can be altered depending on the phases of the migraine cycle [26], we hypothesised that differences between VSS patients and controls in respect of IAF would be more pronounced in the subgroup of subjects without migraine.

## Results

Both groups were matched for age (mean age 33 years, t = 0.8721, *p* = 0.8721, 95% confidence interval [CI] = -7.0581 to 6.0105, degrees of freedom [df] = 40, standard deviation [sd] = 10.4764), sex (male-to-female ratio of 13:8) and migraine (14 subjects had comorbid migraine per group). Additional characteristics of the groups can be found in Table 2.

### Power spectral density in the alpha band (alpha PSD)

The Kruskal-Wallis-test revealed a significant difference in the alpha PSD between the VSS and control groups across the electrodes O1, O2, P9, P10, P3, P4, Pz, T5, T6, T4, and T3 (Kruskal-Wallis-test; *p* = 0.042). In respect of the primary hypothesis, the post-hoc analysis (Mann-Whitney-U-test) did not show significant difference in alpha PSD over the occipital electrodes (O1 *p* = 0.13 and O2 *p* = 0.06 ).

**Fig. 2:**
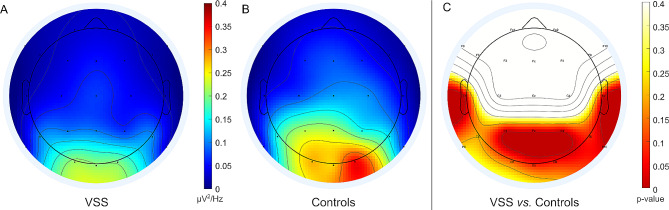
Median power spectral density in the alpha band in patients with visual snow syndrome (VSS) [µV^2^/ Hz] (**A**) and controls (**B**). Distribution of uncorrected *p*-values obtained from the Wilcoxon-Mann-Whitney between the two groups. The regions under the electrodes not tested are white (**C**)

The median PSD was higher in the control group across all electrodes **(**Table 3; Fig. 2). We observed significant differences in the alpha PSD over P10 corresponding to the posterior temporal/basotemporal region on the right side (*p* = 0.009, adjusted *p* = 0.048), bilateral and central parietal regions corresponding to electrodes P3 (*p* = 0.015, adjusted *p* = 0.048), P4 (*p* = 0.015, adjusted *p* = 0.048), Pz (*p* = 0.024, adjusted *p* = 0.048) and midtemporal regions T4 (*p* = 0.026, adjusted *p* = 0.048) and T3 (*p* = 0.021, adjusted *p* = 0.048).

**Table 3:** Difference in alpha power spectral density (PSD) between patients with VSS and controls (post-hoc Wilcoxin-Mann-Whitney, corrected for multiple comparisons using the false discovery rate (FDR) approach)

Electrode	Median VSS	Median cortols	IQR VSS	IQR controls	*p*-Value	*p*-Value (FDR)
P3	0.095	0.221	0.145	0.327	0.015	**0.048**
Pz	0.095	0.217	0.169	0.417	0.024	**0.048**
P4	0.079	0.157	0.126	0.250	0.014	**0.048**
P10	0.031	0.074	0.035	0.160	0.009	**0.048**
P9	0.036	0.049	0.060	0.118	0.222	0.222
O1	0.211	0.279	0.392	0.687	0.128	0.156
O2	0.197	0.352	0.378	0.604	0.064	0.101
T4	0.021	0.040	0.025	0.071	0.026	**0.048**
T3	0.023	0.040	0.021	0.052	0.021	**0.048**
T5	0.099	0.161	0.197	0.326	0.187	0.205
T6	0.101	0.141	0.169	0.324	0.076	0.105

Bold values denote statistical significance

### Individual alpha peak frequency (IAF)

In respect to IAF over the occipital electrodes (O1 and O2), there was no significant difference between the groups (O1: *p* = 0.339, median VSS = 10.613 Hz, median controls = 10.076 Hz, O2: *p* = 0.597, median VSS = 10.132 Hz, median controls = 10.131 Hz).

**Fig. 3:**
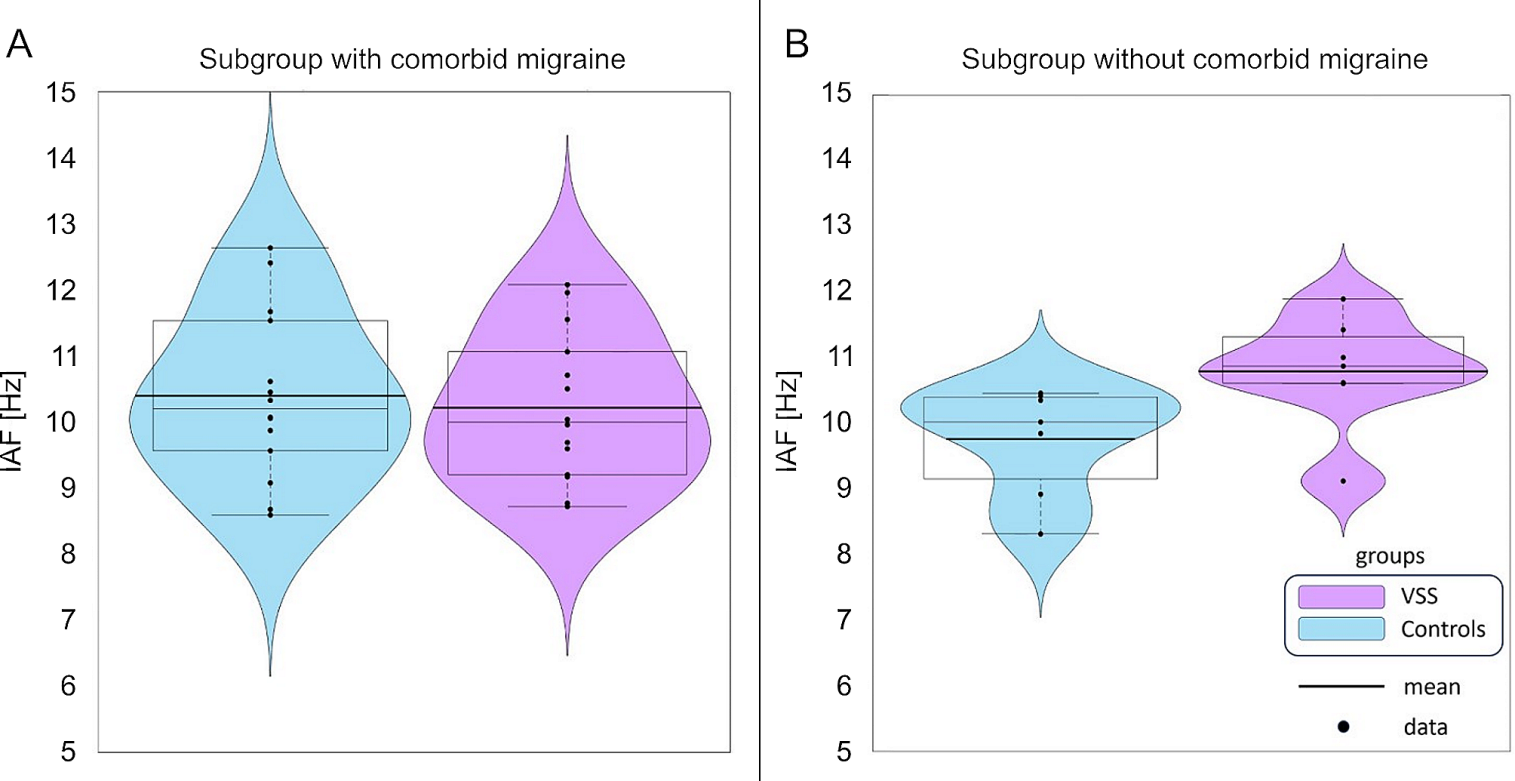
Distribution of the peak individual alpha frequency (IAF) over electrode O1 stratified after presence (in **A**) or absence (in **B**) of comorbid migraine

There was no difference between the VSS group and controls when stratifying for comorbid migraine (Kruskal-Wallis-test *p* = 0.3). Consequently, no further statistical analyses were carried out. Exploratorily, Fig. 3 shows that the IAFs were more widely distributed in the subgroups with migraine. In the subgroups without migraine (*n* = 7 per group), there were notably higher IAFs in the VSS group, not mediated by age (two-sample t-test: *p* = 0.827 for the group without migraine; *p* = 0.800 for the migraine group).

## Discussion

In this study, we investigated the alpha rhythm as potential biomarker for VSS with a focus on alpha PSD and the IAF over the primary and secondary visual cortices.

### Power spectral density in the alpha band (alpha PSD)

In our primary hypothesis, we expected the most significant differences in alpha PSD between the groups to be observed over the primary visual cortex (V1). Although the median alpha PSD over both occipital electrodes is lower in the VSS group, this difference does not reach statistical significance. Based on our data, we must, therefore reject our primary hypothesis. This finding is inconsistent with our initial assumptions, especially considering previous MRI-volumetric studies that have demonstrated increased cortical thickness in the V1 region [7] and electrophysiological evidence indicating a disinhibition in this cortical region. The latter was suggested by studies conducted by Yildiz et al. [13] and Luna et al. [12] which reported reduced habituation of the p-100 response (which corresponds to the afferent visual pathways/V1). Furthermore, a decreased phosphene threshold was demonstrated using transcranial magnetic stimulation (TMS), which could indicate cortical hyperresponsivity in this brain area [13].

In a second, exploratory step, we analysed the alpha PSD over the parietal and temporal electrodes positioned approximately over several brain regions that have been conceptualized as part of the ventral visual pathway (occipitotemporal to the anterior temporal regions) [35] and the dorsal visual pathway (connecting the occipitoparietal cortex to the posterior inferior parietal cortex) [36]. Here, our data shows significant differences between the groups.

The ventral visual pathway, often referred to as the “What pathway”, plays a crucial role in processing representations of object qualities. It is involved in the recognition and identification of visual stimuli [37]. In contrast, the dorsal visual pathway was initially characterised as the “Where pathway”, primarily implicated in visuospatial processing [36]. These pathways are not entirely distinct but rather form interconnected networks. There are interconnections between higher visual areas of both pathways and feedback connections towards the primary visual cortex, contributing to the overall processing and integration of visual information [38].

### Right inferior posterior temporal lobe (P10)

The electrode located posterior infratemporal on the right (P10) is presumed closest to the posterior basotemporal areas, including the lingual and the fusiform gyrus [39, 40]. These areas are part of the secondary visual cortex within the ventral pathway and are located at the border of V1.

Lesion studies have highlighted the central role of these brain areas in the processing and recognising complex visual stimuli [41]. Furthermore, photophobia is a main symptom of VSS and was present in all subjects of our cohort (Table 1). A potential bias from this is worth discussing at this point. Denuelle et al. established a correlation between photophobia in individuals with migraine and an elevation in cerebral blood flow during visual stimulation in secondary visual brain regions, including the bilateral lingual gyri andthe cuneus. In addition to VS, the very high prevalence of photophobia might therefore contribute to our EEG-findings in inferior temporal and parietal brain regions [42].

It is also noteworthy that lesions in posterior temporal brain areas resulting from acute strokes can induce symptoms similar to visual snow (VS) [3].

The lateralization observed in our data (significant on the right side, not significant on the left side), aligns well with imaging findings in patients with VSS showing FDG-PET hypermetabolism with matching cortical volume increase in the visual cortex at the junction of the right lingual and fusiform gyrus [9]. This is of paramount importance since it brings together metabolic, structural, lesional and functional data underscoring the relevance of this region for VSS.

### Midtemporal (T3, T4)

We observed significant differences between the groups in the bilateral temporal regions under the T4 and T3 electrodes. Anatomically this probably encompasses the middle temporal gyrus [43] which plays a crucial role in various functions such as motion perception, together with area V5 located posteriorly/inferior [44], visual integration [45] but also language and auditory processing [43, 46]. In VSS increased grey matter volume in the right midtemporal gyrus has been shown using MRI volumetry [9]. Our study underscores the relevance of this region on an electrophysiological level.

The electrodes are also located close to the auditory brain areas. Based on previous studies that showed a high prevalence of tinnitus in patients with VSS [29], we suspect that tinnitus would be more prevalent within the VSS group, although this was not systematically assessed in our study. Similar to VSS, the reduction of inhibitory alpha modulation has also been hypothesised to play a relevant role in the generation of tinnitus [47].

### Parietal (Pz, P3, P4)

We further identified significant differences between the groups over the bilateral posterior parietal electrodes (P3, P4) and the parieto-occipital area (Pz). These regions are approximately located over hubs of the dorsal visual stream, as mentioned above [43]. These higher-order visual areas within the dorsal stream are reciprocally connected to the primary visual areas and other parts of the secondary visual cortex [36, 48, 49].

### Individual alpha peak frequency (IAF)

In our study, there was no significant difference observed in the IAF when comparing the entire groups. Considering the non-significant outcome of the Kruskal-Wallis-test, we did not perform supplementary statistical analyses. However, we did (descriptively) observe a trend within the subgroup of patients without migraine, where the VSS group exhibited a faster alpha rhythm. This observation stands in contrast to our initial hypothesis. Nevertheless, we think that this merits exploration in future studies.

Migraine has a known influence on IAF, characterized by a reduction in peak frequency with increasing attack frequency and duration. Fluctuations in power and frequency occur throughout different phases of the migraine cycle, which should be taken into consideration when planning studies involving patients VSS and migraine [26].

### Thalamic dysrhythmia or disturbance in top-down-modulation between areas of the visual cortex?

To date the generation of the cortical alpha rhythm remains a topic of discussion. Previous studies using fMRI and PET have shown increased blood flow in the thalamus and brainstem (indicating activation) and decreased blood flow in cortical areas (indicating reduced brain activity) correlating with the alpha rhythm in the resting state, suggesting the thalamus/brainstem as the primary generator of this pattern [52, 53].

In VSS and migraine, the thalamus has been suspected to play a key role in the disturbances of sensory filtering [54, 55]. This was supported by Strik et al. demonstrating a correlation between thalamic T1-signal alterations and VSS symptoms [56].

However, a recent investigation by Halgren et al. using intracranial electrodes in epilepsy patients, revealed that alpha oscillations in the thalamus are preceded by propagation from anterosuperior areas of the secondary visual cortex towards V1. This suggests that cortical oscillators may serve as the primary sources of the alpha rhythm [57]. Furthermore, this was supported by MEG findings which have identified the parietoocipial area as an origin of the cortical alpha rhythm [58].

Taking these findings into consideration, two models would explain our findings: (1) a disturbed inhibitory top-down modulation, with a weakened alpha rhythm generated over secondary visual areas or (2) a decrease in power of the alpha rhythm generated in the thalamus/thalamocortical loops resulting in a spatially more restricted distribution over visual cortex areas.

Further research in this field, incorporating advanced methodologies of EEG source localization or dynamic causal modelling (DCM), would be valuable for gaining deeper insights into these mechanisms.

### Limitations

This study has several limitations. First, despite recording the EEGs in our EEG laboratory using consistent equipment and electrode montages, there remains variability from the setting in clinical routine, such as EEG recordings being conducted at different times of the day, patients suffering from different comorbidities and taking medications that could potentially have an effect on the EEG. Especially centrally acting drugs like antidepressants or anti-seizure medications, which about 25% of participants in both groups are taking, might affect the EEG Further, we did not systematically assess the presence of non-visual VSS symptoms, such as tinnitus and thus cannot exclude that some EEG-findings are related to these symptoms instead of the visual symptoms.

Matching participants precisely for psychiatric disorders or medications was not feasible in this study.

Additionally, the symptoms characterizing visual snow syndrome likely lie on a spectrum within the general population [59]. Although none of the participants in our control group had diagnosed VSS, the absence of VSS symptoms in controls could not be verified due to the retrospective study design.

Similarly, we were unable to consider the influence of different phases of the migraine cycle on the EEGs, as retrospective information on this was not available. Also, it was not documented consistently in our files, if the participants suffered from migraine with or without aura. However, we do not assume a substantial bias for the following reasons: (i) Both groups were thoroughly matched for migraine. We therefore expect all subjects being randomly in the various phases of the migraine cycle, and the effect of the migraine cycle on our findings would be averaged out to some extent. (ii) In the smaller subgroup of patients and controls without comorbid migraine, i.e. in migraine-free participants without migraine cycle, we have observed a trend of a lower alpha PSD over all occipital, temporal, and parietal electrodes in the VSS group that was not significant most likely due to the low sample size (*n* = 7 per group; data not shown). (iii) We have revisited the original EEG files to look at the technician’s comments. The protocol in the EEG lab requires the technicians regularly inquiring participants’ well-being and symptoms at the beginning of the EEG and during the recording. We identified one control group participant who mentioned “a light headache” during recording and exhibited lower alpha PSD in EEG and therefore would have reduced the sensitivity of our analysis. No further subject reported headaches or aura symptoms.

## Clinical or therapeutic implications

Our findings reveal a decreased alpha PSD in several regions of the secondary visual cortex, specifically the temporal and parietal areas. These findings are in line with previous electrophysiological and imaging studies and might therefore contribute to our understanding of the underlying disease mechanisms. Beyond that, our findings might have implications also for migraine. Migraine is the most common comorbidity affecting more than half of patients with VSS and both might have shared pathophysiological mechanisms, such as hyperexcitabilty of the visual cortex and the involvement of thalamocortical pathways [60]. Photophobia is a prevalent symptom in both disorders and was linked to hyperactivity in basotemporal brain areas (especially the lingual gyrus) in persons affected by migraine [42]. Importantly for the results of our study, the alpha rhythm during auras was shown to be desynchronized as well [61]. With these parallels in mind, our findings might therefore also bear implications for visual symptoms related to migraine in general and hold potential therapeutic implications. First, the alpha rhythm can be a target for intervention. It has been demonstrated that the alpha rhythm can be modulated and even entrained using techniques such as transcranial alternating current stimulation (tACS) [62] or transcranial magnetic stimulation(TMS) [63]. Additionally, techniques like biofeedback [64] and meditation [65] may also enhance endogenous alpha rhythms. Second, at the level of neurotransmitters, there is growing evidence that serotonergic pathways, especially thalamocortical projections might lead to an imbalance in inhibitory or excitatory modulation of the visual cortex [66–68]. Here, pharmacological studies to examine effects on VSS and visual symptoms in migraine using the EEG as potential markers might be an interesting way forward [66].

## Conclusion

In our pilot study involving EEG data from 21 patients with visual snow syndrome (VSS) and 21 matched controls, we found a reduced alpha PSD over brain areas belonging to the secondary visual cortex in the VSS group compared to the controls. We also observed a tendency toward higher IAF in VSS, when we excluded participants with comorbid migraine.

Since the primary hypotheses did not yield significant results, all other analyses conducted were exploratory. Our findings should be replicated in follow-up studies, in particular in subgroups without migraine or in patients matched for migraine phases.

These findings underscore the relevance of several temporal and parietal brain areas for the pathophysiology of VSS. This probably includes the lingual and fusiform gyrus, which, based on previous imaging findings, might be relevant for the generation of the VS phenomenon.

## Data Availability

The data supporting this study’s findings and the scripts are available from the corresponding author. Data can only be shared in a pre-processed and anonymised format.

## Abbreviations

VSS: Visual Snow Syndrome
PSD: Power Spectral Density
IAF: Individual Alpha Frequency
EEG: Electroencephalography
